# Construction and validation of a machine learning based prognostic prediction model for children with traumatic brain injury

**DOI:** 10.3389/fped.2025.1581945

**Published:** 2025-05-19

**Authors:** Yongwei Wei, Jiandong Wang, Yu Su, Fan Zhou, Huaili Wang

**Affiliations:** Department of Pediatrics, The First Affiliated Hospital of Zhengzhou University, Zhengzhou, Hennan, China

**Keywords:** traumatic brain injury, machine learning, prediction model, subgroup analysis, children

## Abstract

**Objective:**

This study aimed to establish a prediction model for the short-term prognosis of children with traumatic brain injury (TBI) using machine learning algorithms.

**Methods:**

The clinical data of children with TBI who were treated in the First Affiliated Hospital of Zhengzhou University were retrospectively analyzed. All children were divided into a modeling group and a validation group. In the laboratory indicators of the modeling group, the least absolute shrinkage and selection operator (LASSO) and multivariate Logistic regression analysis were used to screen out the independent influencing factors of poor prognosis in TBI, and a laboratory indicator model (LIM) was established. The risk scores of all patients were calculated. Then, the risk scores and other indicators were used to construct an extended prediction model through the extreme gradient boosting (XGBoost) algorithm. The discrimination, calibration, and clinical utility of the model were evaluated, and the extended model was explained using SHAP analysis. Finally, a subgroup analysis was performed using the risk scores to assess the robustness of the laboratory indicator model.

**Results:**

Among the laboratory indicators, lactate dehydrogenase (LDH), N-terminal pro-B-type natriuretic peptide (NT-proBNP), hydrogen ion concentration index (pH), hemoglobin (Hb), serum albumin (Alb), and C-reactive protein to albumin ratio (CRP/Alb) were the independent influencing factors for the prognosis of children with brain injury. The extended model demonstrated excellent predictive performance in both the modeling and validation populations. SHAP analysis showed the contribution values of the Glasgow Coma Scale (GCS), the laboratory indicator model, the location of the head hematoma, the pupillary light reflex, and the injury severity score in the prediction of the overall patient prognosis. The subgroup analysis showed that there were differences in the risk scores of children with different GCS scores, pupillary light reflexes, and head hematoma locations, and there were also differences in the prognosis between the high-risk score group and the low-risk score group within them.

**Conclusion:**

The extended model can accurately predict the prognosis of TBI patients and has strong clinical utility. The core model has good stratification ability and provides an effective risk stratification and personalized patient management tool for clinicians.

## Introduction

Traumatic brain injury (TBI), a common type of accidental injury in children, is one of the leading causes of severe disability and death among children (1). A sampling survey in hospitals from six provinces in central China found that among 231,162 hospitalized children from 2011 to 2020, there were 15,807 TBI patients, accounting for 6.84% (2). The global burden of disease study reported that the global age standardized incidence rate of TBI was 369/100,000 in 2016 and 346/100,000 in 2019 (3). After traumatic brain injury, children are more prone to epilepsy and other complications than adults (4). Objective and accurate assessment of the condition and prognosis of TBI is the foundation of TBI treatment. The prognosis of TBI is related to multiple factors, including clinical features, laboratory test results, and neuroimaging findings.

The clinical manifestations of TBI patients include headache, dizziness, altered level of consciousness, neurological dysfunction, etc. (5). The Glasgow Coma Scale (GCS) is commonly used clinically to assess the level of consciousness of TBI patients and evaluate their condition and prognosis. In addition, in actual clinical work, children with TBI often have injuries in other parts of the body, which can also affect the prognosis of the children. The Injury Severity Score (ISS) is used clinically to assess the overall severity of trauma in patients (6, 7). However, the GCS score and ISS score are highly influenced by the subjective experience of the evaluators, and patients with the same score at admission often have different clinical prognoses. Therefore, it is necessary to combine laboratory indicators reflecting systemic metabolism and organ function and imaging examinations reflecting head injury conditions to judge the prognosis. Arslan et al. reviewed 161 patients with moderate to severe TBI in the ICU of Istanbul Kanuni Sultan Suleyman Training and Research Hospital of Istanbul Health Sciences University in Turkey from June 2020 to June 2022 and found that the systemic immune-inflammation index (SII), neutrophil - lymphocyte ratio (NLR), and platelet - lymphocyte ratio (PLR) at admission had predictive value for the prognosis of severe TBI patients (8). However, the prediction models based on laboratory indicators are easily affected by testing methods and the selection of different cut-off values, and their universality is poor (5, 9, 10). Therefore, the predictive role of clinical manifestations and imaging data on patient prognosis also needs to be considered. Head CT is currently the most commonly used imaging examination method for children with TBI (11). Researchers have developed many CT scores to assess the condition and prognosis of TBI patients, and the increase in scores is closely related to the risk of death (12, 13). A retrospective study of 250 TBI patients showed that the Marshall, Rotterdam, and NIRIS scoring systems provided good predictions of mortality and outcomes, and subarachnoid hemorrhage was the most common CT finding in death cases (14). However, imaging findings cannot reflect secondary damages such as delayed intracerebral hemorrhage and often require repeated examinations, increasing the radiation harm to patients (15). Therefore, establishing a prognosis prediction model based on multimodal data of patients can comprehensively reflect the complex pathophysiological state of children.

Most traditional TBI prognosis models are constructed based on the assumption of linear relationships and fail to fully consider the nonlinear relationships and interaction effects among variables, resulting in low prediction accuracy of the models (16, 17). Based on the above deficiencies, researchers have begun to seek new methods to more accurately and efficiently grasp these complex relationships. XGBoost (Extreme Gradient Boosting) belongs to the supervised learning algorithm in machine learning (18). It is an ensemble algorithm based on gradient boosting decision trees, with powerful computational efficiency and nonlinear modeling ability. It is mainly used to solve regression and classification problems and has been widely used in risk assessment, prognosis prediction, survival analysis, and other tasks in the medical field. It is increasingly regarded as a viable alternative to traditional linear models (such as logistic regression or Cox regression) for predicting various clinical outcomes (19–22). A retrospective study of 1,123 elderly TBI patients showed that the use of machine learning algorithms to predict the mortality of elderly patients with traumatic brain injury was significantly better than the traditional Logistic algorithm (23). A retrospective study of 1,200 TBI patients by Matsuo et al. showed that XGBoost was significantly better than the logistic regression model in predicting severe disability/vegetative state (24).

Although there have been several previous studies on the prognosis prediction of TBI patients using machine learning, there is a lack of interpretable analysis of the models. SHAP (Shapley Additive exPlanations) is a method used to explain the output of machine learning models. It is based on the Shapley value in cooperative game theory and provides model explanations by calculating the contribution of each feature to the model prediction result. Deeply exploring how changes in feature values affect the model prediction output is crucial for understanding machine learning models (25).

Considering that laboratory indicators are related to the overall physiological state and disease process of patients, the model based on laboratory indicators can perform risk stratification of patients' clinical features. A modeling study of biomarker levels in patients with acute myocardial infarction found that the level of high-sensitivity troponin T (hs - cTnT) had important predictive value for the short-term prognosis of patients. Then, combining the clinical features of patients such as Killip classification (a clinical classification reflecting the degree of heart failure) with the hs - cTnT level could divide patients into different risk groups (26). Therefore, in this study, after establishing a laboratory indicator model, risk stratification of clinical features was performed to improve the practicability of the model.

This study intends to collect the clinical data of children with TBI in the Pediatric Intensive Care Unit (PICU) of the First Affiliated Hospital of Zhengzhou University. Firstly, a Logistic core prediction model containing only laboratory indicators will be established. Secondly, an extended prediction model of XGBoost algorithm for the short-term prognosis of children with TBI will be established. Then, an interpretable method will be applied to enhance the readability of the model. Finally, LIM will be used to perform risk stratification on all patients.

## Methods

### Study design

This retrospective study collected 532 patients with TBI admitted to the Pediatric Intensive Care Unit (PICU) of the First Affiliated Hospital of Zhengzhou University from June 2019 to June 2024. The patients were randomly divided into a modeling group and a validation group at a ratio of 7:3, and were further classified into a good prognosis group and a poor prognosis group based on the prognosis of the children 6 months after the injury.

### Participants

The inclusion criteria were as follows: (1) aged under 18 years; (2) a clear history of head trauma; (3) diagnosed with TBI within 24 h after injury based on symptoms, signs, or imaging (head CT or MRI); (4) completion of all examinations and tests within 24 h after TBI and before surgical or other drug treatment measures; (5) first admission with basically complete clinical data.

The exclusion criteria were as follows: (1) death within 24 h of admission; (2) a history of multiple previous brain injuries, such as craniotomy, spinal cord injury, or penetrating head injury; (3) suffering from severe underlying diseases, such as heart, liver, or kidney insufficiency; (4) having clinical manifestations of hematological diseases, malignant tumors, chronic inflammation, or acute infection before injury; (5) recent use of steroids, immunosuppressants, antiplatelet agents, or oral anticoagulants.

### Study procedures

(1) Basic information: age, gender, past medical history, time of injury, place of injury (Road/Home/Other), cause of injury (Traffic injury/Falling - related injuries/Other), injured area (Single site or Multiple sites).
(2) Clinical information: vomiting after injury, disturbance of consciousness after injury, seizure after injury, pupillary light reflex (PR), presence or absence of coagulation disorder, Glasgow Coma Scale (GCS), Injury Severity Score (ISS).
(3) Imaging findings: location of hematoma in the head (LH) (epidural/subdural/subarachnoid/intracerebral/mixed), presence or absence of skull fracture, open or closed.
(4) Laboratory indicators: pH, blood sugar, chloride ion, white blood cells, red blood cells, hemoglobin (Hb), platelets, neutrophils, lymphocytes, monocytes, alanine aminotransferase, aspartate aminotransferase, albumin, globulin, total protein, serum creatinine, creatine kinase, creatine kinase isoenzyme, lactate dehydrogenase (LDH), N-terminal pro-brain natriuretic peptide (NT - pro BNP), procalcitonin, C-reactive protein (CRP), interleukin-6, neutrophil-to-lymphocyte ratio (NLR), platelet-to-lymphocyte ratio (PLR), lymphocyte-to-monocyte ratio (LMR), C-reactive protein to albumin ratio (CAR).
(5) Study outcome: Telephone follow-up was conducted on the 180th day after admission. According to the Glasgow Outcome Scale-Extended, Pediatric Revision (GOSE-peds) score, patients were divided into a good prognosis group and a poor prognosis group.
  The good prognosis group included: GOSE score of 8, indicating full recovery; GOSE score of 7, indicating good recovery (with slight functional deficits but close to full recovery); GOSE score of 6, indicating moderate mild disability (partial recovery of occupational or social functions); GOSE score of 5, indicating mild moderate disability (independent living with limited ability).
  The poor prognosis group included: GOSE score of 4, indicating moderate severe disability (requiring significant assistance but partial independence); GOSE score of 3, indicating severe disability (completely dependent on others, requiring 24-h care); GOSE score of 2, indicating vegetative state; GOSE score of 1, indicating death.
(6) Missing value handling: The VIM package in R language was used to appropriately fill in the missing values after fully considering the distribution of variable values.

### Statistical analysis

**Figure 1:**
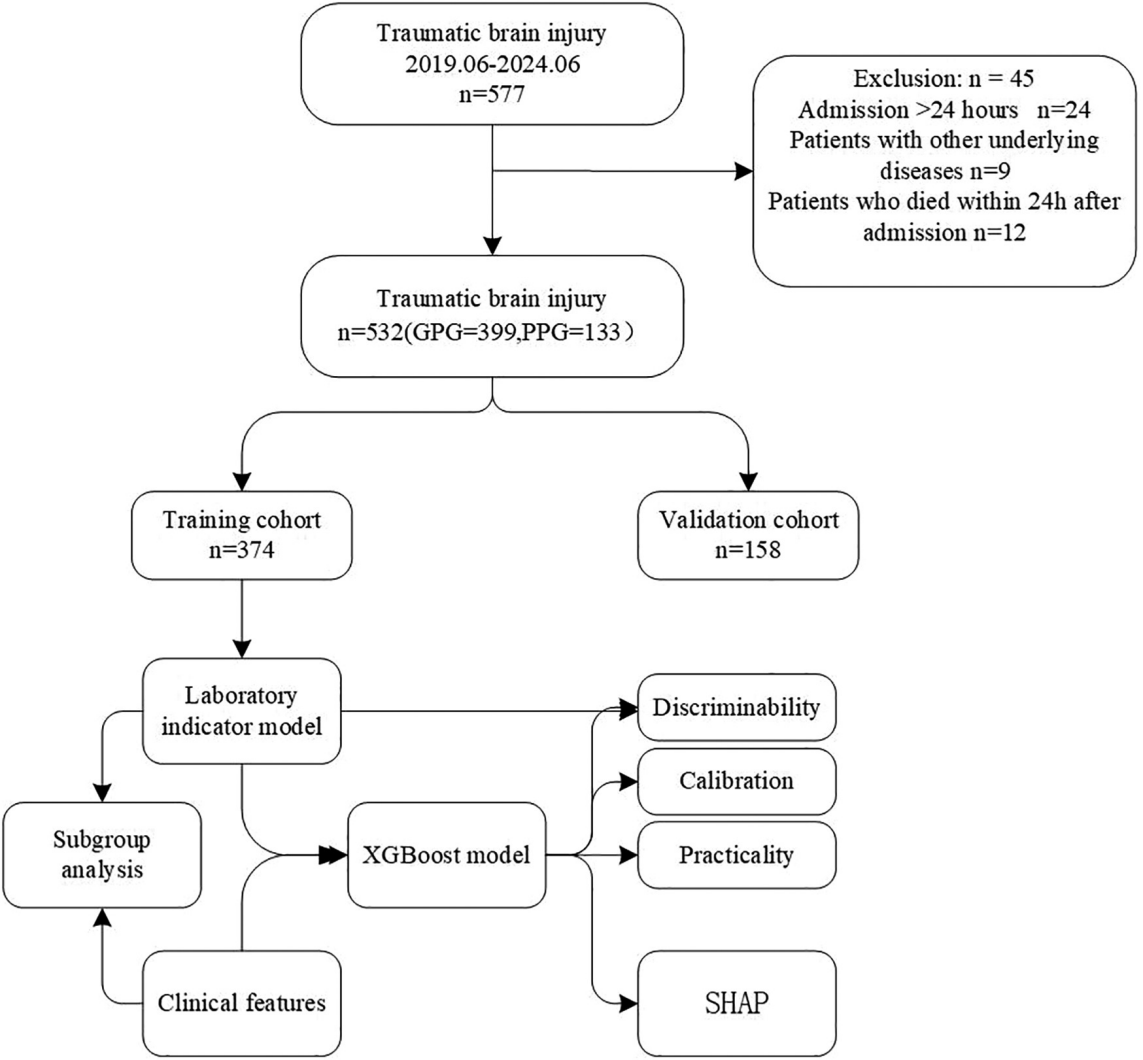
Flow chart of the study.

All statistical analyses, model construction, validation, and interpretation in this study were based on R software (version 4.4.2). The significance level was set at *α* = 0.05, and a *P*-value less than 0.05 was considered statistically significant. Firstly, to improve the predictive performance of the model, the cut-off values of all continuous variables were calculated and dichotomized. Categorical variables were expressed as percentages, and comparisons between groups were analyzed using the chi-square test or Fisher's exact test. LASSO regression was applied to 27 laboratory indicators in the modeling population to screen for factors influencing poor prognosis of TBI. The screened variables were then included in multivariate Logistic regression to further screen for independent influencing factors of poor prognosis of TBI and establish a core prediction model. The ROC curve was drawn and the AUC was calculated to evaluate the discrimination of the prediction model. Then, the risk scores of all patients were calculated based on the established laboratory model. Five important features were screened out through XGBoost together with the risk scores and other indicators to construct an XGBoost prediction model. To visualize the model for clinical use, the five important features screened out by XGBoost were modeled using Logistic regression and a nomogram was drawn. To test the effectiveness of the model, a confusion matrix was first drawn to evaluate the robustness of the XGBoost model. Then, the ROC curve was drawn and the AUC was calculated to evaluate the discrimination of the model. The calibration curve was drawn to evaluate the calibration of the model, and the clinical decision curve was drawn to evaluate the clinical utility of the model. SHAP was used to explain the XGBoost model. A waterfall plot was drawn to present the contribution values and directions of each feature in promoting the model prediction results. A swarm plot and a bar plot of feature importance were drawn to macroscopically display the feature importance and SHAP values of the entire model. A dependence plot was drawn to show the relationship between individual feature values and SHAP values. Finally, a stratified analysis of the prognostic risk of patients was performed based on the Glasgow Coma Scale, pupillary light reflex, and location of head hematoma using the risk scores, as shown in Figure 1.

### Ethics

All procedures in this study complied with the principles of the institutional and/or national research committee and the Declaration of Helsinki. The study was approved by the Scientific Research and Ethics Committee of the First Affiliated Hospital of Zhengzhou University (Ethics Approval No.: 2023-KY-1242-001).

## Results

The data were randomly divided into a training set and a validation set at a ratio of 7:3. There were 374 patients in the training group and 158 patients in the validation set, as shown in Table 1.

**Table 1:** Baseline characteristics of traumatic brain injury patients stratified by prognostic Status and cohort.

Characteristics	Whole	Prognostic status		*p*	Cohort		*p*
Groups	Patients	Favorable	Unfavorable		Training	Validation	
*n*	(*n* = 532)	(*n* = 399)	(*n* = 133)		(*n* = 374)	(*n* = 158)	
Gender, *n* (%)				0.39			0.841
Female	170 (32)	123 (31)	47 (35)		49 (31)	121 (32)	
Male	362 (68)	276 (69)	86 (65)		109 (69)	253 (68)	
Age, *n* (%)				0.144			0.292
≤4.5 year	239 (45)	187 (47)	52 (39)		77 (49)	162 (43)	
>4.5 year	293 (55)	212 (53)	81 (61)		81 (51)	212 (57)	
Injured area, *n* (%)				0.012			1
Single site	323 (61)	255 (64)	68 (51)		96 (61)	227 (61)	
Multiple sites	209 (39)	144 (36)	65 (49)		62 (39)	147 (39)	
Causes of injury, *n* (%)				<0.001			0.049
Other	33 (6)	29 (7)	4 (3)		15 (9)	18 (5)	
Falling - related injuries	251 (47)	203 (51)	48 (36)		65 (41)	186 (50)	
Traffic injuries	248 (47)	167 (42)	81 (61)		78 (49)	170 (45)	
Site of injury, *n* (%)				0.008			0.253
Other	46 (9)	41 (10)	5 (4)		17 (11)	29 (8)	
Home	200 (38)	157 (39)	43 (32)		52 (33)	148 (40)	
Road	286 (54)	201 (50)	85 (64)		89 (56)	197 (53)	
Emesis, *n* (%)				<0.001			0.821
No	321 (60)	222 (56)	99 (74)		97 (61)	224 (60)	
Yes	211 (40)	177 (44)	34 (26)		61 (39)	150 (40)	
Disturbance of awareness, *n* (%)				<0.001			0.168
No	220 (41)	210 (53)	10 (8)		73 (46)	147 (39)	
Yes	312 (59)	189 (47)	123 (92)		85 (54)	227 (61)	
Post - traumatic seizure, *n* (%)				<0.001			1
No	446 (84)	362 (91)	84 (63)		132 (84)	314 (84)	
Yes	86 (16)	37 (9)	49 (37)		26 (16)	60 (16)	
Pupil reflex, *n* (%)				<0.001			0.397
Normal	396 (74)	360 (90)	36 (27)		122 (77)	274 (73)	
Abnormal	136 (26)	39 (10)	97 (73)		36 (23)	100 (27)	
GCS, *n* (%)				<0.001			0.052
≤9	179 (34)	64 (16)	115 (86)		43 (27)	136 (36)	
>9	353 (66)	335 (84)	18 (14)		115 (73)	238 (64)	
ISS, *n* (%)				<0.001			0.625
≤15	364 (68)	339 (85)	25 (19)		111 (70)	253 (68)	
>15	168 (32)	60 (15)	108 (81)		47 (30)	121 (32)	
Coagulation dysfunction, *n* (%)				<0.001			0.355
Normal	285 (54)	238 (60)	47 (35)		90 (57)	195 (52)	
Abnormal	247 (46)	161 (40)	86 (65)		68 (43)	179 (48)	
Location of hematoma, *n* (%)				<0.001			0.934
Epidural hematoma	270 (51)	257 (64)	13 (10)		83 (53)	187 (50)	
Subdural hematoma	60 (11)	46 (12)	14 (11)		15 (9)	45 (12)	
Subarachnoid hematoma	65 (12)	37 (9)	28 (21)		19 (12)	46 (12)	
Intracerebral hematoma	102 (19)	44 (11)	58 (44)		30 (19)	72 (19)	
Multiple hematomas	35 (7)	15 (4)	20 (15)		11 (7)	24 (6)	
Cranial CT, *n* (%)				<0.001			0.609
Normal	122 (23)	117 (29)	5 (4)		39 (25)	83 (22)	
Abnormal	410 (77)	282 (71)	128 (96)		119 (75)	291 (78)	
Skull fracture, *n* (%)				0.023			0.552
No	231 (43)	185 (46)	46 (35)		65 (41)	166 (44)	
Yes	301 (57)	214 (54)	87 (65)		93 (59)	208 (56)	
Craniocerebral injury, *n* (%)				0.042			0.845
Open	412 (77)	318 (80)	94 (71)		121 (77)	291 (78)	
Closed	120 (23)	81 (20)	39 (29)		37 (23)	83 (22)	
PH, *n* (%)				0.07			0.111
≤7.429	290 (55)	227 (57)	63 (47)		95 (60)	195 (52)	
>7.429	242 (45)	172 (43)	70 (53)		63 (40)	179 (48)	
Bs, *n* (%)				<0.001			0.264
≤6.85	343 (64)	275 (69)	68 (51)		108 (68)	235 (63)	
>6.85	189 (36)	124 (31)	65 (49)		50 (32)	139 (37)	
Cl, *n* (%)				<0.001			0.453
≤109.5	450 (85)	352 (88)	98 (74)		137 (87)	313 (84)	
>109.5	82 (15)	47 (12)	35 (26)		21 (13)	61 (16)	
WBC, *n* (%)				1			0.324
≤10.91	4 (1)	3 (1)	1 (1)		0 (0)	4 (1)	
>10.91	528 (99)	396 (99)	132 (99)		158 (100)	370 (99)	
RBC, *n* (%)				<0.001			0.6
≤3.715	135 (25)	68 (17)	67 (50)		43 (27)	92 (25)	
>3.715	397 (75)	331 (83)	66 (50)		115 (73)	282 (75)	
Hb, *n* (%)				<0.001			0.888
≤105.45	161 (30)	85 (21)	76 (57)		49 (31)	112 (30)	
>105.45	371 (70)	314 (79)	57 (43)		109 (69)	262 (70)	
Plt, *n* (%)				<0.001			0.514
≤249.5	181 (34)	110 (28)	71 (53)		50 (32)	131 (35)	
>249.5	351 (66)	289 (72)	62 (47)		108 (68)	243 (65)	
Neu, *n* (%)				0.03			0.311
≤8.735	233 (44)	186 (47)	47 (35)		75 (47)	158 (42)	
>8.735	299 (56)	213 (53)	86 (65)		83 (53)	216 (58)	
Lym, *n* (%)				0.016			0.076
≤1.2	169 (32)	115 (29)	54 (41)		41 (26)	128 (34)	
>1.2	363 (68)	284 (71)	79 (59)		117 (74)	246 (66)	
Mon, *n* (%)				0.009			0.106
≤0.715	317 (60)	251 (63)	66 (50)		103 (65)	214 (57)	
>0.715	215 (40)	148 (37)	67 (50)		55 (35)	160 (43)	
ALT, *n* (%)				<0.001			0.689
≤14.5	227 (43)	200 (50)	27 (20)		70 (44)	157 (42)	
>14.5	305 (57)	199 (50)	106 (80)		88 (56)	217 (58)	
AST, *n* (%)				<0.001			0.228
≤38.5	280 (53)	241 (60)	39 (29)		90 (57)	190 (51)	
>38.5	252 (47)	158 (40)	94 (71)		68 (43)	184 (49)	
TP, *n* (%)				<0.001			0.451
≤63.25	193 (36)	116 (29)	77 (58)		53 (34)	140 (37)	
>63.25	339 (64)	283 (71)	56 (42)		105 (66)	234 (63)	
Alb, *n* (%)				<0.001			0.372
≤40.25	192 (36)	103 (26)	89 (67)		52 (33)	140 (37)	
>40.25	340 (64)	296 (74)	44 (33)		106 (67)	234 (63)	
Glb, *n* (%)				0.001			0.599
≤18.95	89 (17)	54 (14)	35 (26)		29 (18)	60 (16)	
>18.95	443 (83)	345 (86)	98 (74)		129 (82)	314 (84)	
Cre, *n* (%)				<0.001			0.444
≤43.5	464 (87)	360 (90)	104 (78)		141 (89)	323 (86)	
>13.5	68 (13)	39 (10)	29 (22)		17 (11)	51 (14)	
CK, *n* (%)				<0.001			0.131
≤208.5	312 (59)	272 (68)	40 (30)		101 (64)	211 (56)	
>208.5	220 (41)	127 (32)	93 (70)		57 (36)	163 (44)	
CKL, *n* (%)				<0.001			0.15
≤22.85	144 (27)	127 (32)	17 (13)		50 (32)	94 (25)	
>22.85	388 (73)	272 (68)	116 (87)		108 (68)	280 (75)	
LDH, *n* (%)				<0.001			0.008
≤349.5	238 (45)	214 (54)	24 (18)		85 (54)	153 (41)	
>349.5	294 (55)	185 (46)	109 (82)		73 (46)	221 (59)	
NT – pro BNP, *n* (%)				<0.001			0.961
≤242.515	405 (76)	330 (83)	75 (56)		121 (77)	284 (76)	
>242.515	127 (24)	69 (17)	58 (44)		37 (23)	90 (24)	
PCT, *n* (%)				<0.001			0.267
≤0.366	350 (66)	302 (76)	48 (36)		110 (70)	240 (64)	
>0.366	182 (34)	97 (24)	85 (64)		48 (30)	134 (36)	
CRP, *n* (%)				<0.001			0.677
≤3.235	312 (59)	269 (67)	43 (32)		90 (57)	222 (59)	
>3.235	220 (41)	130 (33)	90 (68)		68 (43)	152 (41)	
IL-6, *n* (%)				<0.001			0.367
≤19.325	282 (53)	241 (60)	41 (31)		89 (56)	193 (52)	
>19.325	250 (47)	158 (40)	92 (69)		69 (44)	181 (48)	
NLR, *n* (%)				0.002			1
≤2.64	126 (24)	108 (27)	18 (14)		37 (23)	89 (24)	
>2.64	406 (76)	291 (73)	115 (86)		121 (77)	285 (76)	
PLR, *n* (%)				0.031			0.14
≤247.195	166 (31)	114 (29)	52 (39)		57 (36)	109 (29)	
>247.195	366 (69)	285 (71)	81 (61)		101 (64)	265 (71)	
LMR, *n* (%)				0.002			0.268
≤3.87	347 (65)	245 (61)	102 (77)		97 (61)	250 (67)	
>3.87	185 (35)	154 (39)	31 (23)		61 (39)	124 (33)	
CAR, *n* (%)				<0.001			1
≤0.08	317 (60)	275 (69)	42 (32)		94 (59)	223 (60)	
>0.08	215 (40)	124 (31)	91 (68)		64 (41)	151 (40)	

Notes: The *p*-values in the above table are calculated by chi-square test.

Abbreviations: GCS, Glasgow coma scale; ISS, injury severity score; PH, PH value; Bs, blood sugar; Cl, chloride ion; WBC, white blood cells; RBC, red blood cells; Hb, hemoglobin; Plt, platelets; Neu, neutrophils; Lym, lymphocytes; Mon, monocytes; ALT, alanine aminotransferase; AST, aspartate aminotransferase; TP, total protein; Alb, albumin; Glb, globulin; Cre, serum creatinine; CK, creatine kinase; CKL, creatine kinase isoenzyme; LDH, lactate dehydrogenase; NT-proBNP, N-terminal pro-brain natriuretic peptide; PCT, procalcitonin; CRP, C-reactive protein; IL-6, interleukin-6;NLR, neutrophil-to-lymphocyte ratio; PLR, platelet-to-lymphocyte ratio; LMR, lymphocyte-to-monocyte ratio; CAR, C-reactive protein to albumin ratio.

### Core model construction and validation

**Figure 2:**
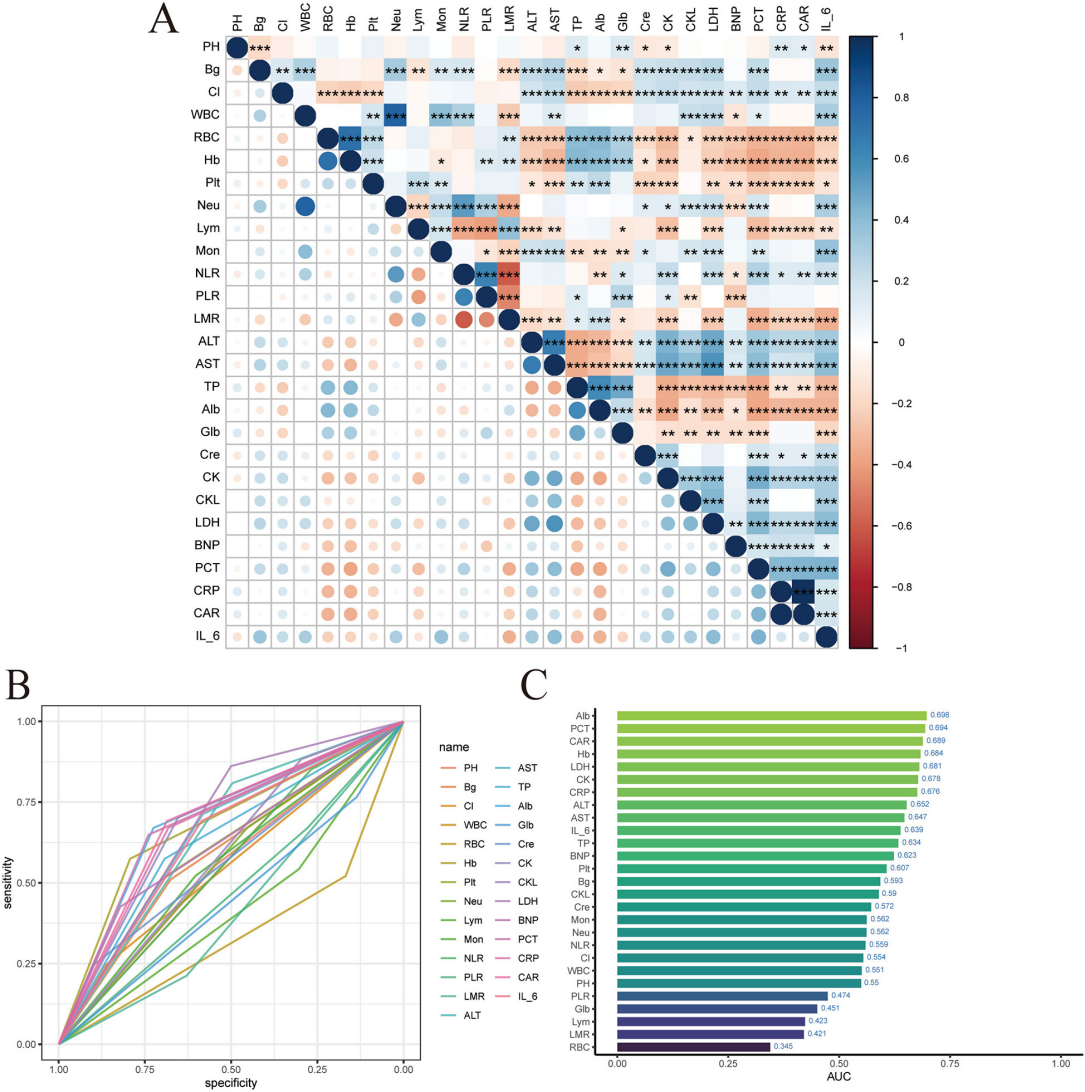
LIM variable filtering. **(A)** Laboratory index correlation heat map. **(B)** Laboratory index ROC. **(C)** Laboratory index AUC value.

Firstly, a heatmap was drawn using the training set data to display the correlations among the 27 laboratory indicators, as shown in Figure 2A. Then, the ROC curves of the 27 variables were drawn to show the predictive performance, as shown in Figure 2B. A histogram was drawn to display the AUC of the 27 variables, as shown in Figure 2C.

**Figure 3:**
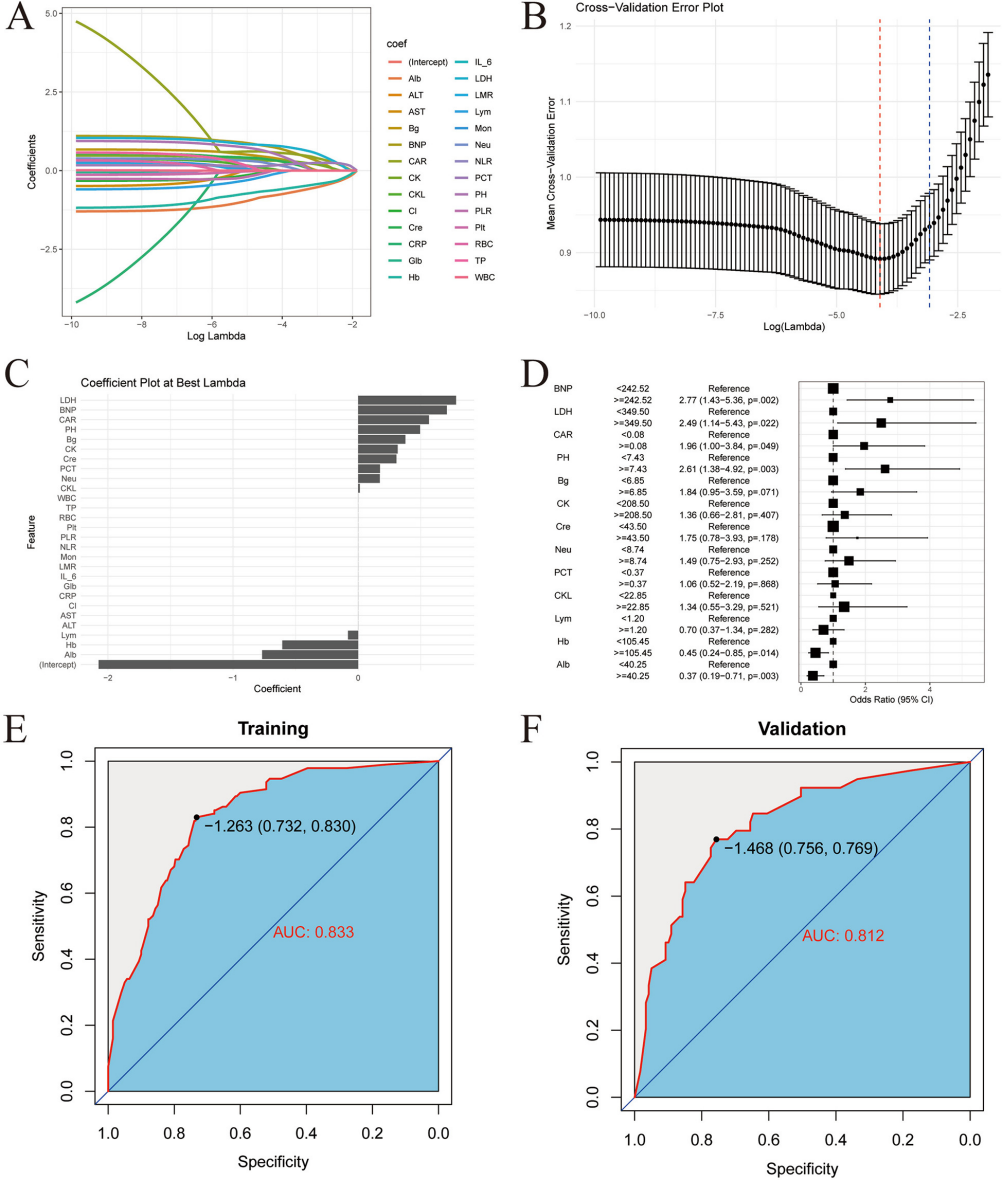
LIM model construction and validation. **(A)** Lasso regression coefficient path map. **(B)** Lasso regression cross validation chart. **(C)** Minimum mean square error time variable. **(D)** Multivariate logistic regression forest map. **(E)** ROC curve of training set. **(F)** ROC curve of validation set.

LASSO regression was used to screen variables among the 27 laboratory indicators in the modeling population. As shown in Figure 3A. To achieve a good model fit, the λ corresponding to the minimum mean square error was selected after cross-validation, as shown in Figure 3B. Through LASSO regression analysis, 13 variables were obtained, namely: pH, Hb, Alb, LDH, NT - pro BNP, CAR, neutrophils, lymphocytes, serum creatinine, creatine kinase, creatine kinase isoenzyme, procalcitonin, and blood glucose, as shown in Figure 3C.

To further screen the independent influencing factors affecting the prognosis of children with TBI, the above 13 variables were included in the multivariate Logistic regression analysis. The results showed that pH, Hb, Alb, LDH, NT – pro BNP, and CAR were the independent influencing factors for poor prognosis in TBI patients, as shown in Figure 3D. A laboratory indicator prediction equation was constructed using the 6 screened independent influencing factors: Y = −1.96 + 1.414X1 + 0.8647X2 + 0.7137X3 + 0.5456X4-0.7328X5-1.0892X6 (X1 represents LDH, X2 represents NT - pro BNP, X3 represents CAR, X4 represents pH, X5 represents Hb, X6 represents Alb).

The risk scores were calculated according to the regression equation, and the ROC of the subjects was drawn, and the AUC was calculated to evaluate the discrimination of the prediction model. The AUC of the training set was 0.833 (95% CI: 0.789–0.877), as shown in Figure 3E. The AUC of the validation set was 0.812 (95% CI: 0.734–0.890), as shown in Figure 3F. This indicates that the core model based on laboratory indicators has good discrimination.

### Extended model establishment and validation

**Figure 4:**
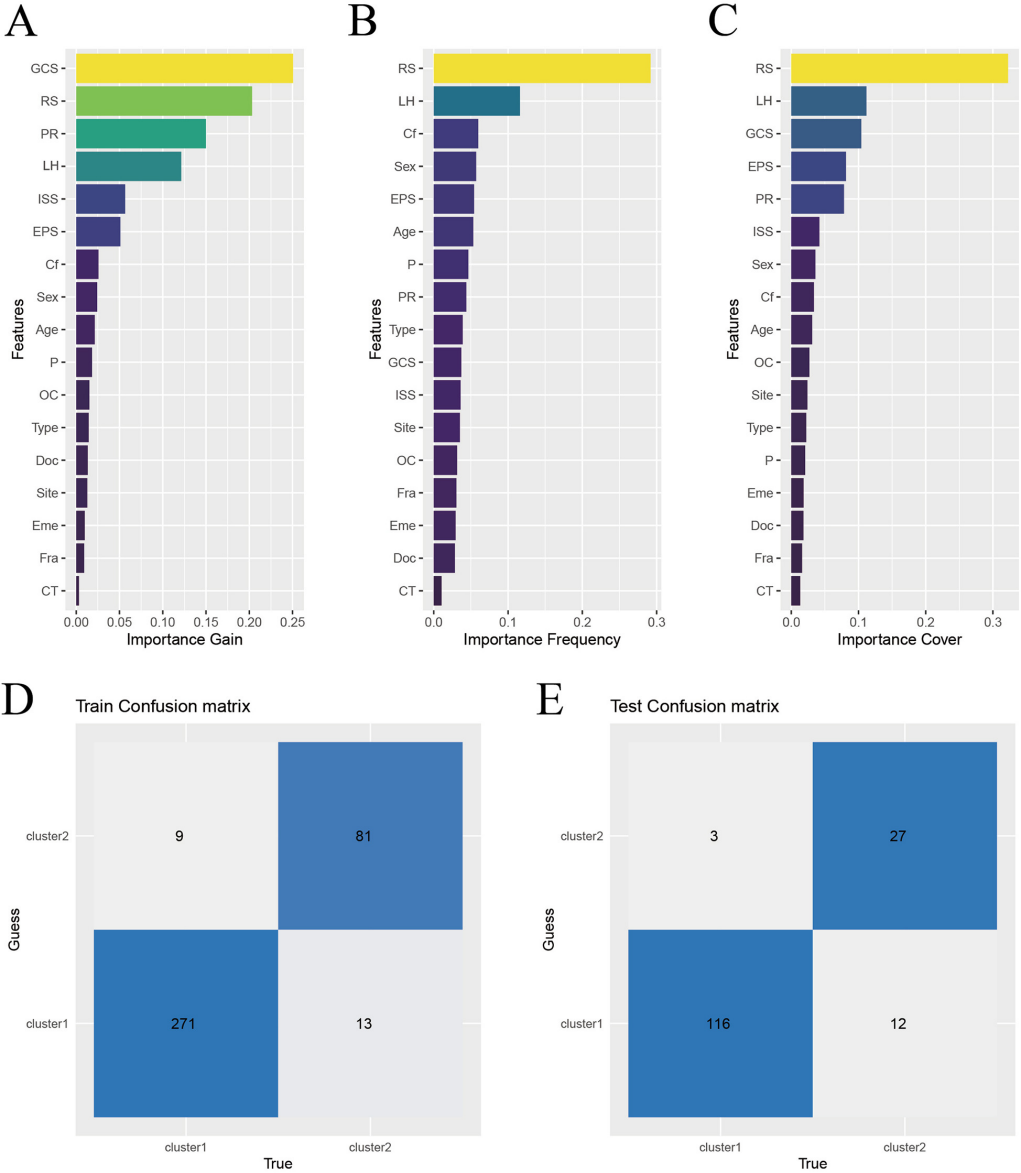
Construction and validation of XGBoost model. **(A)** Characteristic gain ordering. **(B)** Ranking of the number of feature splitting points. **(C)** Sorting of sample number of characteristic sub nodes. **(D)** Training set confusion matrix. **(E)** Validation set confusion matrix. Abbreviations: RS, risk score; PR, pupillary light reflex; LH, location of hematoma; EPS, early post-traumatic seizures; Cf, coagulation function; P, injured area (single site or multiple sites); OC, craniocerebral injury (open or close); Type, causes of injury (traffic injury/falling - related injuries/other); Eme: vomiting after injury; Doc, disturbance of consciousness after injury; Site, place of injury (Road/Home/Other); Fra, Skull fracture; CT, Cranial CT.

XGBoost analysis was used to rank the important features of the risk scores and other indicators in the modeling population. The top 5 in the order of importance were: GCS, LIM, pupillary light reflex, hematoma location, and ISS, as shown in Figure 4A. These 5 indicators were included in the XGBoost algorithm to establish a machine learning model.

To test the effectiveness of the model, the confusion matrix showed that the overall accuracy of the XGBoost model in the training set reached 94.1%, as shown in Figure 4D. The overall accuracy in the test set was 90.5%, as shown in Figure 4F. This indicates that the model has good classification ability.

**Figure 5:**
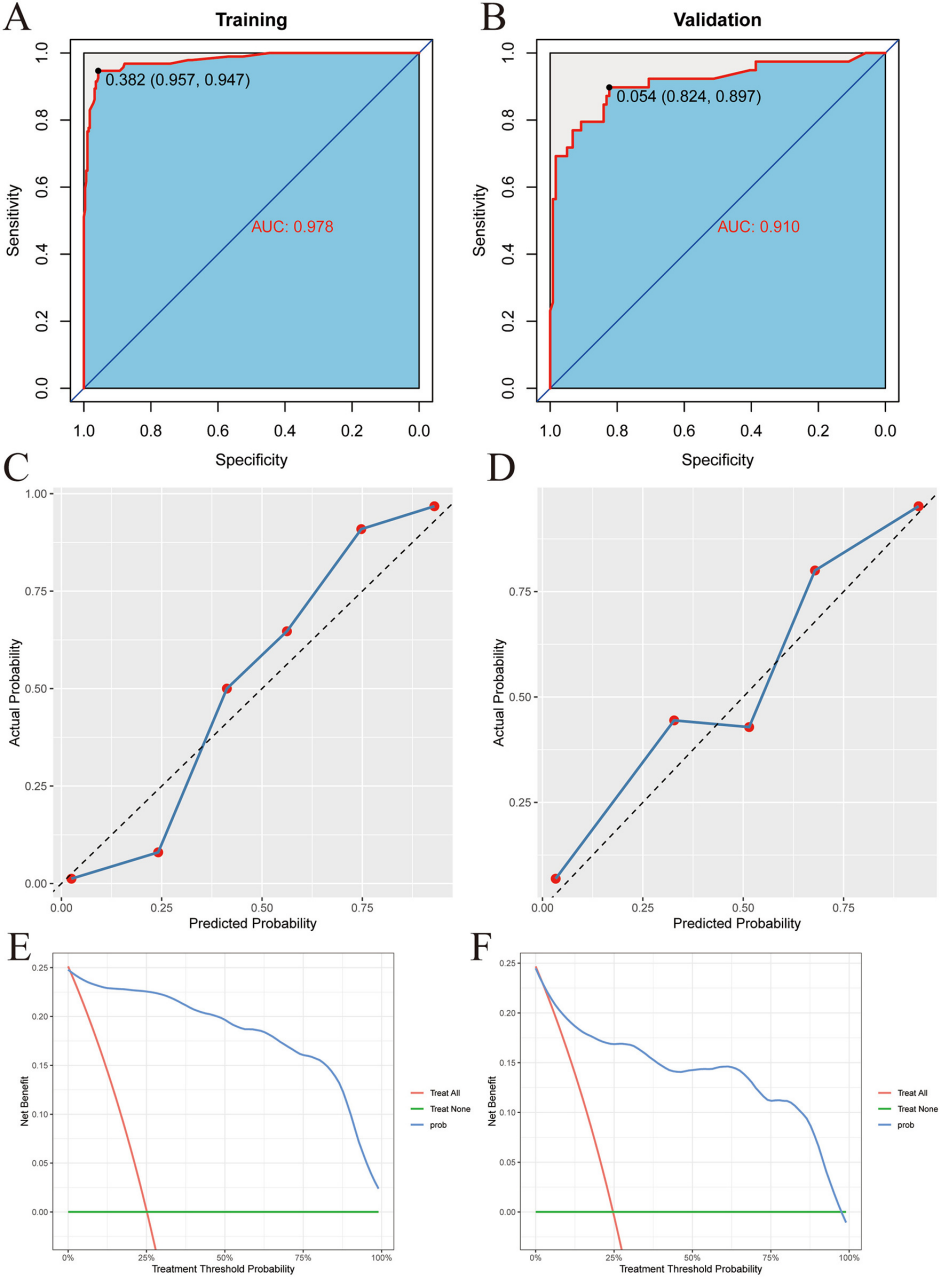
XGBoost model validation. **(A)** Training set ROC. **(B)** Validation set ROC. **(C)** Training set calibration curve. **(D)** Validation set calibration curve. **(E)** Training set decision curve. **(F)** Validation set decision curve.

Then, the ROC curves were drawn in the training set and the validation set respectively, and the AUC was calculated to evaluate the discrimination of the prediction model. The AUC of the training set was 0.978 (95% CI: 0.962–0.994), as shown in Figure 5A. The AUC of the validation set was 0.910 (95% CI: 0.847–0.974), as shown in Figure 5B. This indicates that XGBoost has good discrimination. The Bootstrap internal resampling method was used to repeat sampling 1,000 times in the modeling group data, and the concordance index (C-index) was 0.925.

Calibration plots were drawn in the training set and the validation set, as shown in Figures 5C,D. The figure shows that the predicted situation of poor prognosis of TBI is basically consistent with the actual situation, indicating that the prediction model has good calibration.

**Figure 6:**
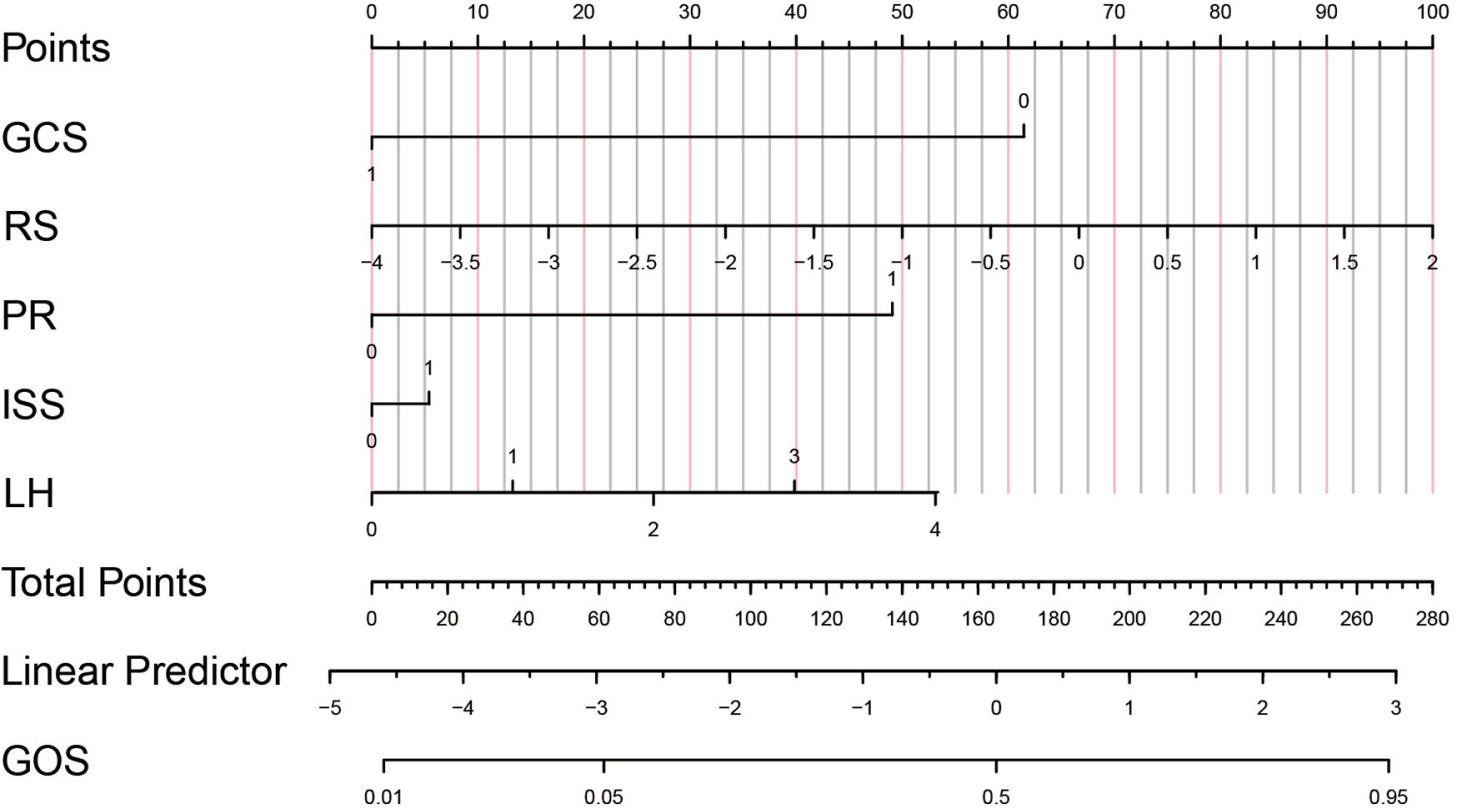
Predictive model of prognosis nomogram of children with TBI. GCS, 1 = GCS ≥9, 0 = GCS <9; RS, Risk score; PR (pupil to light reflex), 1 = positive, 0 = negative; ISS (Injury Severity Score), 1 = ISS ≥15, 0 = ISS<15; LH (location of hematoma in the head): 0 = Epidural hematoma, 1 = Subdural hematoma, 2 = Subarachnoid hematoma, 3 = Intracerebral hematoma, 4 = Multiple hematomas.

Finally, decision curves were drawn in the training set and the validation set, as shown in Figures 5E,F. The abscissa represents the threshold probability, and the ordinate represents the net benefit of patients receiving treatment. The horizontal green line above the abscissa represents the net benefit of assuming that all patients are not treated, the red line represents the net benefit of assuming that all patients are treated, and the blue line represents the net benefit of using the XGBoost model to guide treatment. This indicates that the prediction model has good clinical utility. To facilitate clinical use, the top 5 important features selected by XGBoost were incorporated into a Logistic regression model to develop a nomogram, as shown in Figure 6.

### SHAP explanation

**Figure 7:**
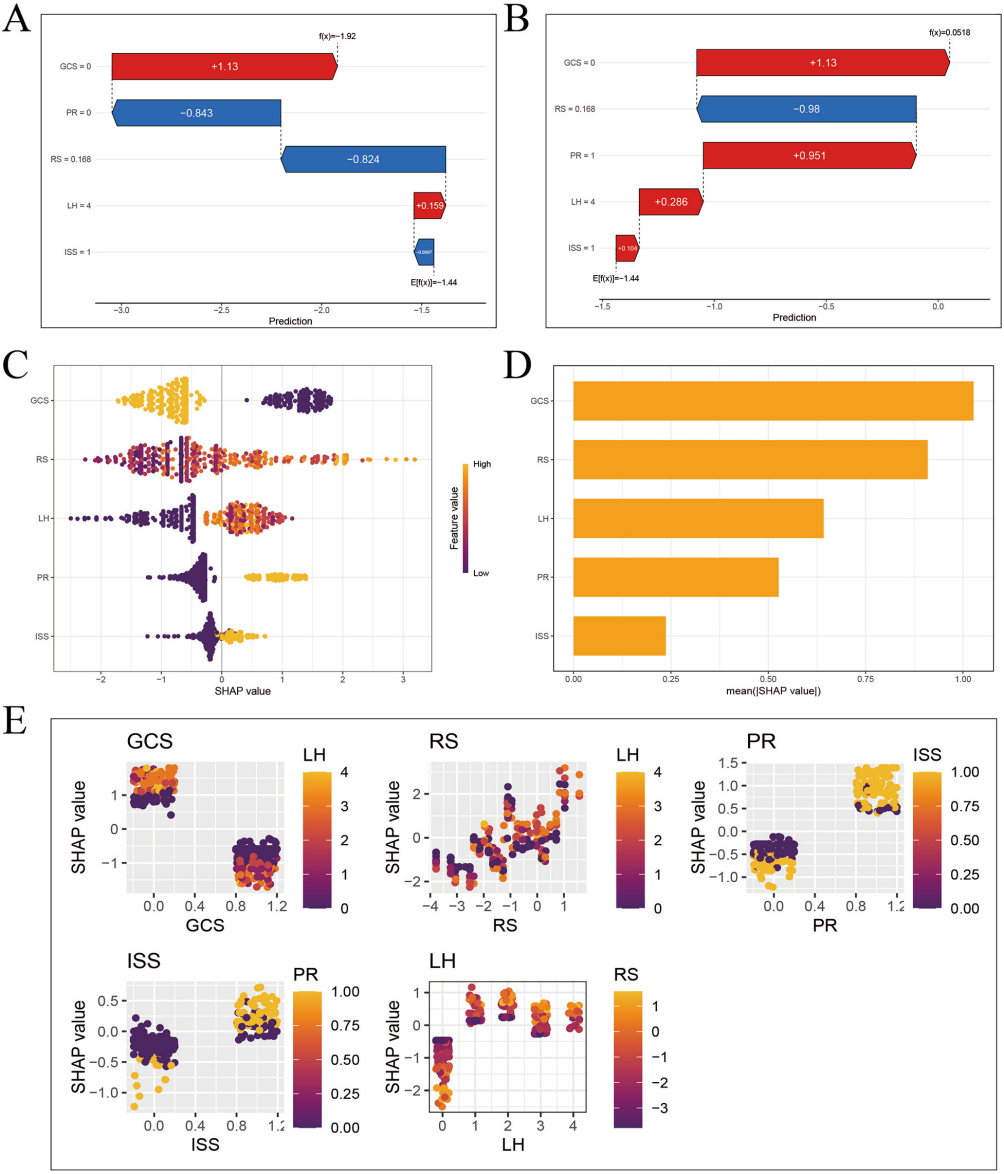
SHAP interpretation of XGBoost model. **(A)** Waterfall diagram of sample 3 (good prognosis). **(B)** Waterfall diagram of sample 4 (poor prognosis). **(C)** Feature importance bee colony graph. **(D)** Feature importance histogram. **(E)** Multivariate partial correlation dependence graph.

To explain the XGBoost model, firstly, a SHAP waterfall plot was drawn to show the contribution values and directions of each feature in affecting the model prediction results. Starting from the baseline value, each feature value pushes the baseline forward or backward. For these two patients, the feature values “GCS”, “LIM” and “PR” contributed the most to the prognosis prediction, as shown in Figures 7A,B.

The SHAP feature importance swarm plot and feature importance bar plot were drawn, and the features were sorted in descending order by feature importance. The horizontal axis represents the Shapley value of the corresponding feature. The feature “GCS” is the most important, as shown in Figures 7C,D.

Finally, the SHAP multivariate dependence plot was drawn to represent the marginal effect of a single feature on the model prediction result, showing the linear, monotonic or more complex relationship between the feature and the Shapley value. Each point in the partial dependence plot represents a sample. The horizontal axis represents the value of the corresponding feature of the sample, and the vertical axis represents the Shapley value of the feature. The lower the GCS score, the higher the Shapley value, and the worse the prognosis of TBI patients. The higher the LIM score, the higher the Shapley value, and the worse the prognosis of TBI patients. The abnormal PR, the higher the Shapley value, and the worse the prognosis of TBI patients. The higher the ISS score, the higher the Shapley value, and the worse the prognosis of TBI patients. The Shapley value of epidural hematoma is significantly lower than that of the other four types of hematoma, and the prognosis is better, as shown in Figure 7E.

Through SHAP explanation, we identified the specific contribution values of important features in the prognosis prediction of different children with TBI, and intuitively demonstrated the complex prediction process of the model.

### Risk stratification

**Figure 8:**
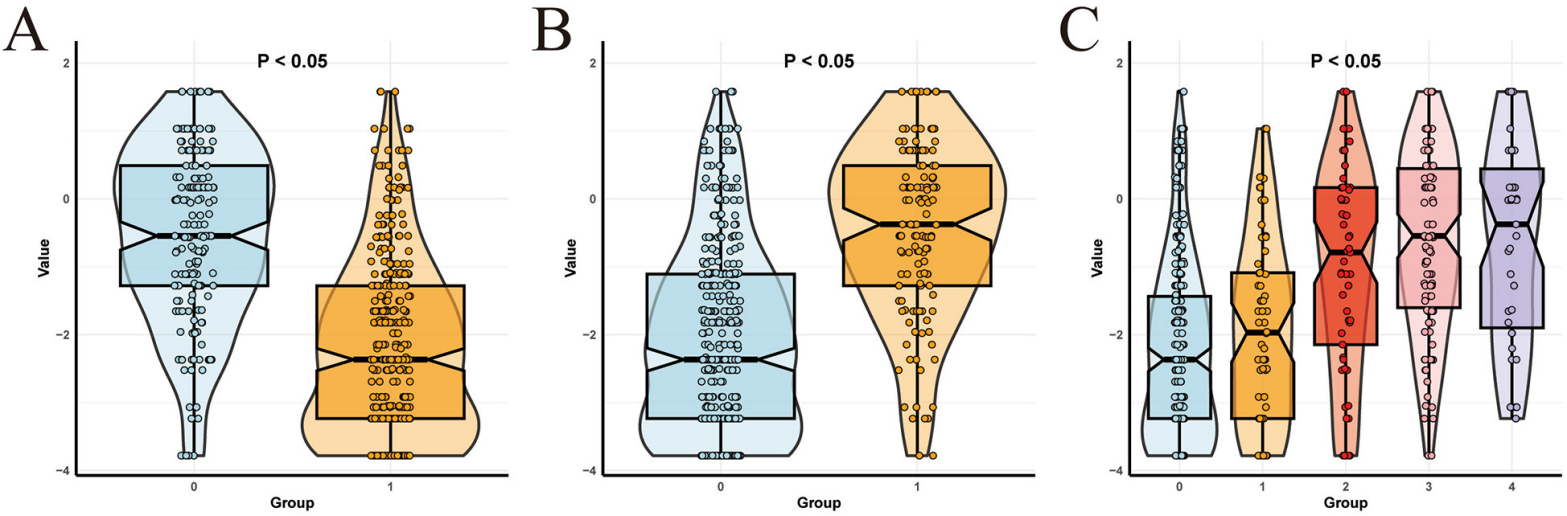
Subgroup analysis. **(A)** Comparison of risk scores between low and high Glasgow score groups. **(B)** Comparison of risk scores between negative and positive pupillary reflex groups. **(C)** Comparison of risk scores of different parts of head hematoma.

To better apply the laboratory indicator model in clinical practice, we used risk scores to group the clinical features by risk. The cut - off value of the risk score calculated in the training set was −1.26, and all patients were divided into a high - risk score group and a low - risk score group. When we compared the risk scores of the high GCS group and the low GCS group, the difference was statistically significant (*P* < 0.001), as shown in Figure 8A. Then, when we compared the prognosis of the high - risk score group and the low - risk score group within the high GCS group and the low GCS group respectively, the difference was statistically significant (*P* < 0.001). Comparing the risk scores of the normal pupil reflex group and the abnormal pupil reflex group, the difference was statistically significant (*P* < 0.001), as shown in Figure 8B. Then, comparing the prognosis of the high-risk score group and the low-risk score group within the normal pupil reflex group and the abnormal pupil reflex group respectively, the difference was statistically significant (*P* < 0.001). Comparing the risk scores of patients with different head hematoma locations, the difference was statistically significant (*P* < 0.001), as shown in Figure 8C. Then, comparing the prognosis of the high-risk score group and the low-risk score group within the epidural hematoma group, the difference was statistically significant (*P* < 0.05). Comparing the prognosis of the high-risk score group and the low-risk score group within the subarachnoid hematoma group, the difference was statistically significant (*P* < 0.001). Comparing the prognosis of the high-risk score group and the low-risk score group within the intracerebral hematoma group, the difference was statistically significant (*P* < 0.001). Comparing the prognosis of the high-risk score group and the low-risk score group within the mixed hematoma group, the difference was statistically significant (*P* < 0.05). This indicates that among patients with the same Glasgow Coma Scale score, pupil reaction, and head hematoma location, patients with higher risk scores have a worse prognosis, which greatly improves the clinical applicability of the laboratory indicator model.

## Discussion

TBI is a common critical illness in pediatrics. Its heterogeneity brings numerous challenges to clinical treatment, and accurate assessment of the condition and prognosis of children with TBI is of great significance for their treatment. In this study, the clinical data of 532 children with TBI who visited the Pediatric Intensive Care Unit (PICU) of the First Affiliated Hospital of Zhengzhou University were collected, including clinical features, laboratory indicators, and imaging findings. Firstly, a laboratory indicator model was established using LASSO-Logistic regression, and the risk scores of all patients were calculated. Then, the XGBoost algorithm of machine learning was used to screen out 5 features with high contribution from the risk scores, clinical features, and imaging findings to establish an XGBoost model, and the model was tested. Since the XGBoost model is a “black box” system and cannot be directly used in clinical practice, the features with high contribution in XGBoost were incorporated into the Logistic regression model in this study and visualized in the form of a nomogram for clinical use. To explore the influence of each feature in the XGBoost model on the outcome index and the accurate contribution of different features in different samples, waterfall plots, importance swarm plots, importance bar plots, and partial correlation dependence plots were drawn based on the SHAP algorithm to explain the XGBoost model. Finally, this study found that the laboratory indicator model can subgroup clinical features such as Glasgow Coma Scale score, pupil reaction, and head hematoma location.

In this study, lactate dehydrogenase passed the LASSO-Logistic regression and was included in the laboratory indicator model as an independent influencing factor, indicating that the increase in lactate dehydrogenase in TBI patients is related to poor prognosis. Lactate dehydrogenase is a glycolytic enzyme widely present in various tissues of the human body. After TBI, lactate dehydrogenase is rapidly released into the intercellular space and finally enters the bloodstream through the damaged blood-brain barrier, resulting in a rapid increase in serum lactate dehydrogenase levels (27). Liu Y et al. found that the level of lactate dehydrogenase in the poor prognosis group of children with TBI was significantly higher than that in the good prognosis group, suggesting that lactate dehydrogenase may be an important indicator for evaluating the severity and prognosis of TBI, which is consistent with the findings of this study (28). In children with TBI, lactate dehydrogenase detection can help evaluate the severity of the injury and the prognosis of the disease.

This study found that an increase in pH value is an independent risk factor for poor prognosis in children with TBI. Studies have shown that under alkalosis conditions, cerebrovascular constriction reduces the oxygen supply to brain tissue, thereby exacerbating the metabolic burden in the damaged area (8). Richter et al. found that inflammatory markers such as GFAP and UCH-L1 were more significant under increased pH conditions, suggesting that systemic alkalosis may enhance the neuroinflammatory response and exacerbate neuronal damage (29). Korhone et al. pointed out in an analysis of abnormal values of blood biomarkers in patients with acute TBI that abnormal changes in pH value may be associated with higher mortality, especially in the presence of other metabolic disorders (30). Chen et al. proposed a model for predicting the prognosis of TBI by combining the inverse shock index (rSIG) with the metabolic state, emphasizing that an increase in pH value may be an indicator of systemic metabolic disorder and is closely related to poor 90-day prognosis (31). In conclusion, the increase in pH value is of great significance in the prognosis assessment of TBI patients. Future studies can further investigate the relationship between the dynamic changes of pH value and long-term functional recovery to improve the prognosis of TBI patients.

This study found that an increase in NT-pro BNP is an independent risk factor for poor prognosis in children with TBI. Ru et al. found that the levels of NT-pro BNP in plasma and cerebrospinal fluid of patients with TBI were significantly increased (32). Richter et al. analyzed the data of 872 patients with moderate to severe TBI and found that patients with increased NT-pro BNP levels had a poor prognosis, especially in patients with mild imaging lesions (Marshall score <3) (29). These studies are consistent with the findings of this study. The mechanism may be that brain injury triggers cardiac dysfunction by exciting the sympathetic nervous system, resulting in an increase in NT-pro BNP levels, suggesting that NT-pro BNP may be a potential marker of the severity of TBI.

This study found that a decrease in albumin is an independent risk factor for poor prognosis in children with TBI. Nayak et al. found that in patients with severe TBI, those with an albumin level lower than 3.5 g/dl at admission had a significantly increased mortality rate and poor functional outcome (33). The study also indicated that albumin is an independent prognostic factor and recommended its inclusion in routine prognostic assessment. Luo et al. found that an albumin level lower than 30 g/L at admission was significantly associated with higher mortality and disability risk in children with TBI (34). Hashim et al. showed that dynamic monitoring of changes in albumin levels (especially in the first 5 days after trauma) is of great significance for predicting 90-day functional recovery in patients with TBI (35). In conclusion, low albumin levels are closely related to poor prognosis, suggesting continuous monitoring for early intervention.

This study found that an increase in CAR level is an independent risk factor for poor prognosis in children with TBI. Dogan et al. studied the predictive role of CAR in intracranial injury in patients with TBI and found that an increase in CAR level was associated with more severe intracranial lesions, especially in patients with cerebral edema or hematoma (36). Wang et al. showed that an increase in CAR is an independent prognostic risk factor and emphasized that incorporating CAR into the comprehensive assessment model of TBI can improve the prediction accuracy (37). A meta-analysis of 5 studies involving 1,040 patients showed that CAR is related to the mortality rate of patients with TBI (38). In conclusion, the CAR ratio is a convenient and comprehensive inflammation-nutrition assessment tool. Its increase may indicate a higher mortality rate and poorer functional outcome, and it has important value in early risk stratification and management strategies.

This study found that a decrease in Hb level is an independent risk factor for poor prognosis in children with TBI. Hifumi et al. found that an early high Hb level (>13.5 g/dl) in patients with severe TBI was significantly associated with a good neurological prognosis at 6 months, while a low Hb level significantly increased the risk of poor prognosis (39). Lee et al. found that a decrease in Hb ratio was an independent factor for predicting poor neurological outcome in infants with TBI. The study further pointed out that Hb level is related to the degree of increased intracranial pressure and cerebral edema (40). Boutin et al. analyzed the influence of the red blood cell transfusion threshold in patients with TBI and found that a lower Hb transfusion threshold (<7 g/dl) was associated with a higher in-hospital mortality rate, indicating that maintaining an appropriate Hb level is of great significance for ICU management (41). In conclusion, low Hb levels are significantly related to poor prognosis in patients with TBI, suggesting that clinical personalized Hb management strategies can be formulated to improve the clinical outcome of patients with TBI.

The above six laboratory indicators constitute the core prediction model of this study. The AUC in the training set is 0.833 (95% CI: 0.789–0.877), and the AUC in the validation set is 0.812 (95% CI: 0.734–0.890), indicating that the core model based on laboratory indicators has good discrimination. Compared with a prognostic model of TBI patients that included 10 indicators including clinical features in a study by Lu et al., with similar discrimination, this study included fewer indicators, had a lower risk of overfitting, and was more convenient for clinical use (42).

The Glasgow Coma Scale is the most important instrumental variable for predicting the prognosis of TBI screened by XGBoost. According to many studies, the Glasgow Coma Scale is an important factor for predicting the prognosis of TBI patients. A Glasgow Coma Scale score lower than 8 is generally considered a sign of poor prognosis (43–46). King et al. showed that a low Glasgow Coma Scale score is not only related to the short-term risk of death but also can effectively predict long-term neurological dysfunction (47). The study analyzed that patients with a low Glasgow Coma Scale score for a long time required more rehabilitation treatment and had a slow neurological recovery process and poor prognosis. A study using machine learning technology to predict the prognosis of adult patients with isolated moderate and severe TBI showed that the most influential factor for model prediction was the Glasgow Coma Scale score, which is consistent with the results of this study (45).

The pupillary light reflex is a sensitive indicator for detecting the integrity of the optic nerve and oculomotor nerve function and the functional state of the midbrain (48). As a non-invasive neurological function assessment method, it has gradually received attention in evaluating the prognosis of brain injury. Trent et al. showed that quantitative pupillometry could predict neurological deterioration in patients with acute TBI, with a specificity of 91.67%, indicating that abnormal PR is an early indicator of neurological deterioration (49). Dengler et al. showed through a study of military TBI patients that pupillometry could be used to dynamically monitor changes in patients' neurological function and effectively predict the duration and severity of symptoms (50). Oddo et al. showed in a multicenter cohort study that PR was closely related to the 6-month prognosis of patients with TBI, and its change trajectory could significantly improve the accuracy of prognosis assessment (51). In addition, studies have shown that the combination of PR and the Glasgow Coma Scale can further improve the prediction effect of the prognosis of TBI, which further supports the universality of the variable structure of this model (52, 53).

Head hematoma is a common pathological manifestation of TBI. The location of the hematoma has a significant impact on the neurological function, mortality rate, and complications of patients with TBI. A retrospective analysis of 14,075 patients with TBI hospitalized in a tertiary hospital in southern Thailand showed that the location types of head hematomas such as acute subdural hematoma and subarachnoid hemorrhage were prognostic factors related to in-hospital mortality and were used together with age, hypotension, antiplatelet drugs, Glasgow Coma Scale score, pupillary light reflex, skull base fracture, acute subdural hematoma, subarachnoid hemorrhage, and midline shift to construct a nomogram (54). A meta-analysis of 10,733 patients with TBI showed that the presence of subdural hematoma with cerebral edema was associated with a worse outcome, and epidural hematoma (EDH) was associated with a better outcome (55). A 5-year, six-center retrospective cohort study showed that the acute onset of chronic isolated subdural hematoma was identified as an independent risk factor for neurosurgical intervention (56).

ISS is a tool for comprehensively assessing the severity of trauma. It mainly considers the injury conditions of different regions of the body (including the head, face, neck, chest, abdomen, limbs, and pelvis). Each region's injury is assigned a score (from 1 to 6 points) according to its severity, and then the sum of the squares of the scores of the three most severely injured regions of the body is calculated to obtain ISS. Because in real life, children with TBI often have concomitant injuries in other parts of the body, this scoring method can comprehensively reflect the overall trauma degree of the children. Roepke et al. showed in a retrospective cohort study that ISS has advantages in multisystem blunt trauma (57). Brown et al. studied the data of children with trauma under 16 years old in the Pennsylvania Trauma Registry in the United States and found that the children's ISS threshold was 25 points, and children with ISS ≥25 points benefited the most from appropriate triage (58).

## Conclusions

This study has some limitations. First of all, after the model is established, only internal validation has been carried out, and other external data are still needed to verify its prediction efficiency. Secondly, this model is based on the data of a single regional research center, and it still needs large sample research based on the data of multi regional research centers to establish a more accurate and efficient prognosis prediction model for children with TBI.

The core model established in this study using LDH, NT Pro BNP, pH, Hb, ALB, car has a good discrimination for the poor short-term prognosis of children with TBI, and provides a powerful risk stratification and personalized patient management tool for clinicians. The extended model based on XGBoost machine learning algorithm has good predictive ability for the prognosis of children with TBI. The developed nomogram can accurately predict the prognosis of patients with TBI, and has strong clinical practicability.

## Data Availability

The datasets presented in this article are not readily available because the data are governed by strict ethical and regulatory restrictions to ensure compliance with patient privacy and confidentiality standards. Requests to access the datasets should be directed to Yongwei Wei, 13087082776@163.com.
